# Epidemiological survey of registered violence cases in covered hospitals of Isfahan Health Center in 2016

**Published:** 2019-08

**Authors:** Hamid Turkzadeh, Alireza Dadaei shahrekordi, Ali Salehi, Seyed Mohsen Fayaz

**Affiliations:** ^*a*^Center Manager - Isfahan Health Center No. 1 - Isfahan University of Medical Sciences, Isfahan, Iran.; ^*b*^Unit for Combating Illness- Isfahan Health Center No. 1 - Isfahan University of Medical Sciences, Isfahan, Iran.; ^*c*^Deputy Health Center- Isfahan Health Center No. 1 - Isfahan University of Medical Sciences, Isfahan, Iran.

## Abstract

**Background:**

Violence should be regarded as ambiguity, which, unlike the consensus in its rejection, has a wide range in human social life. Human beings have been damaged and harmed by violence for many years, however, human beings have not always tolerated or violated violence. There are moments in history that one has been informed about his violence towards his fellow man and seeks to find a way to overcome it. Today, what has become the cause of the concern of various communities is the increasing extent of violence, less the day that the media devote several columns to their pages or parts of their news to describe violent acts. Every day, we witness the perpetration of violent acts at the individual and social levels. Increasing the rate of murder, assault and rape, conflict, the spread of wars and internal conflicts, ethnic and religious conflicts, and social rebellions all reflect the fact that the level of inclination to violence is increasing and patterns change. A decade ago, when it was talked about violence, peoples’ minds tended to be focused on certain types of violence, such as violence against women or children, but today they are talking about one or more specific types of violence Not meaningful. Today, what focuses on everyone’s attention is the spread of violence in all its forms and manifestations. The aim of this study was to investigate epidemiologic cases of violence referred to hospitals affiliated to Isfahan Health Center No. 1 in 1395.

**Methods:**

This was a retrospective descriptive study. The statistics were collected by examining the accident and accident forms and analyzed using SPSS software package.

**Results:**

Of 21239 incidents, 1,000 (4.7%) were related to violence and the fifth highest accident rate occurred in urban areas. The injuries included 81.9% of men and 18.1% of women. 97% of violence occurred in urban areas and 3% in rural areas. In terms of violence, most cases occurred in 42.9% of streets and 23.4% at home. The highest percentage of violence was observed in the age groups of 20-29 years old (25.7%), 30-39 (20.7%), 10-19 years (15.7%), 40-49 years (10.1%) and 50- 59 years (7.1%). More than 99% of the violence was treated and only 5 People reported death.

**Conclusions:**

The analysis of the above statistics shows that violence among incidents has a relatively high percentage that requires a special look as one of the components of social health. However, the existence of violence at young ages and ages of active and active society of bells A serious threat to mental health in society. Since most violence has occurred in urban areas and there are several factors such as stress, economic problems, lifestyles and other cultural and economic factors, more research is needed to accurately identify causes and factors of violence and find solutions to prevent them. This is underlined by this article.

**Keywords:**

Violence, Active age group, Prevention

